# One-Month Outcomes of Cases Receiving Ticagrelor after Percutaneous Coronary Intervention; a Case Series 

**Published:** 2020-03-30

**Authors:** Mohammad Hasan Namazi, Farzam Saemifard, Mehdi Pishgahi

**Affiliations:** 1 Interventional Cardiology department, Shahid Modarres Heart Center, Shahid Beheshti University of Medical Sciences, Tehran, Iran.; 2 Interventional Cardiology Department, Shohadaye Tajrish hospital, Shahid Beheshti University of Medical Sciences, Tehran, Iran.

**Keywords:** Ticagrelor, patient outcome assessment, drug-related side effects and adverse reactions, percutaneous coronary intervention, platelet aggregation inhibitors

## Abstract

**Introduction:**

Ticagrelor is the first reversibly binding oral P2Y12 receptor antagonist that can block ADP-induced platelet aggregation. This study aimed to describe one-month follow-up findings of cases undergoing ticagrelor therapy after percutaneous coronary intervention (PCI).

**Methods:**

This case series was performed on acute coronary syndrome (ACS) patients who were candidates for PCI and received aspirin plus ticagrelor after PCI. Patients were followed for one month and their outcomes were described.

**Results:**

156 cases with the mean age of 59.74 ± 9.24 years were studied (63% male). 45 (28.8%) cases complained of dyspnea (39 cases with mild and 6 cases with severe dyspnea). Bleeding occurred in 4 (2.5%) cases (intra-cranial hemorrhage (ICH) in one, hematuria in two, and skin hemorrhage in one case). There were no cases with bradycardia or thrombosis. One (0.6%) patient developed drug hypersensitivity reaction, which manifested as skin rash. The use of drug was stopped in 10 (6.4%) cases due to severe dyspnea (n= 6), ICH (n=1), skin rash (n=1), and concomitant left ventricular (LV) clot (n=2).

**Conclusion:**

The most important finding of one-month ticagrelor consumption were dyspnea, bleeding, and hypersensitivity reaction. No case of bradycardia and stent thrombosis was detected. In our study , iranian population has more susceptibility to dyspnea than PLATO result. The rate of drug discontinuation in this series of cases was 6.4 %.

## Introduction

Antiplatelet and anti-coagulants are used to prevent stent thrombosis and repeated acute coronary events in patients undergoing primary percutaneous coronary intervention (PPCI) (1, 2). According to existing guidelines, rather than using clopidogrel (P2Y12), prescription of more potent agents from this family, such as ticagrelor is recommended (3, 4). Ticagrelor is a potent drug with class I of recommendation for acute coronary syndrome (ACS) patients (5). Possible adverse effects include bleeding and dyspnea, which are mainly mild and do not require intervention (6, 7).

Ticagrelor is the first reversibly binding oral P2Y12 receptor antagonist that can block ADP-induced platelet aggregation (8). Unlike thienopyridines, which irreversibly binds to P2Y12 receptor throughout the platelet’s lifetime, ticagrelor can bind to the receptor reversibly and exhibits a rapid onset and offset of effect, corresponding to drug exposure levels (4, 9).

Since this drug has recently been introduced in pharmacies in Iran and there are no studies on its adverse effects such as bleeding, dyspnea, bradycardia, and etc. (10). This study aimed to describe the one-month follow-up findings of cases that underwent ticagrelor therapy after percutaneous coronary intervention (PCI).

## Methods

***Study design and setting***

This case series was performed on ACS patients who were candidates for PPCI and were referred to the emergency department of Modarres Hospital, Tehran, Iran, from September 2018 to September 2019 (one year). Based on the hospital protocol, aspirin plus clopidogrel or aspirin plus ticagrelor were prescribed for patients as anti-platelet aggregation agents. In the present study, one-month outcomes of cases that consumed aspirin plus ticagrelor are described. The study protocol was approved by local Ethics committee of Modarres Hospital and also Ethics committee of Shahid-Beheshti University of Medical Sciences (Code: IR.SBMU.MCP.REC.1397.602). 

***Participants***

ACS patients who were candidates for PPCI and consumed aspirin plus ticagrelor were enrolled using consecutive sampling. Patients with end-stage renal disease (ESRD), sensitivity to the studied drugs, history of coagulation disorders and history of fibrinolytic therapy in the past 48 hours were excluded. 

Patients received aspirin plus ticagrelor loading dose (180 mg; Brilinta from AstraZeneca Co.) with maintenance of 90 mg ticagrelor twice a day plus 80 mg ASA daily. They were followed-up for one month monitoring possible complications.

***Data analysis:***

Data analysis was carried out using SPSS (version 19.0) software. The numerical data were presented as mean ± standard deviation and categorical ones were shown as frequency and percentage. 

## Results

***Baseline characteristics of studied cases***

156 cases with the mean age of 59.74 ± 9.24 (ranging 37 to 78) years were studied (63% male). ST segment elevation myocardial infarction (STEMI) (42%), unstable angina (36%), and non-STEMI (22%) were the most common diagnoses, respectively. Diabetes mellitus (34%) and hypertension (28%) were the most frequent underlying diseases of studied cases.

***Outcomes***

45 (28.8%) cases complained of dyspnea (39 cases with mild and 6 cases with severe dyspnea). Bleeding occurred in 4 (2.5%) cases (intra-cranial hemorrhage (ICH) in one, hematuria in two, and skin hemorrhage in one case). There were no cases with bradycardia or thrombosis. One (0.6%) patient developed drug hypersensitivity reaction, which manifested as skin rash. The use of drug was stopped in 10 (6.4%) cases due to severe dyspnea (n= 6), ICH (n=1), skin rash (n=1), and concomitant left ventricular (LV) clot (n=2). 

## Discussion

The most important findings after one month of ticagrelor consumption in this series were dyspnea (28.8%), bleeding (2.5%), and hypersensitivity reaction (0.6%). No case of bradycardia and stent thrombosis was detected. The rate of drug discontinuation in this series of cases was 6.4 %.

Ticagrelor has potential to change the standard of drug therapy for ACS patients as shown in Platelet inhibition and patient outcomes (PLATO) trial, but long-term studies are required to further evaluate its efficacy and safety in these patients (11). Ticagrelor is the first reversibly binding oral P2Y12 receptor antagonist that blocks ADP-induced platelet aggregation (12). Risk of mild to moderate dyspnea and mostly asymptomatic ventricular pauses were observed in phase II trials of Ticagrelor (13).

In PLATO study, at 12 months, the primary end point included death from vascular causes, myocardial infarction, or stroke, which had occurred in 9.8% of patients receiving versus 11.7% of those receiving clopidogrel (14). However, there were no positive thrombotic cases in our study. Additionally, in PLATO study (15), ticagrelor showed a higher rate of major bleeding, which was not related to the procedure, compared to clopidogrel. Ticagrelor caused significantly more instances of fatal intracranial bleeding, but fewer fatal bleeding of other types. In our study, there was only one case with intracranial bleeding.

The study by Levin et al. (16) showed improved survival and reduction in major cardiovascular events with ticagrelor consumption. Kang et al. (17) reported that cardiovascular event rates are higher in Asians, but bleeding rates are similar and, the effects of ticagrelor versus clopidogrel are not significantly different between Asians and non-Asians with respect to the primary outcome and efficacy. 

Overall, this study revealed that ticagrelor is an effective drug but it should be used with caution as there are major concerns regarding its side effects such as severe dyspnea and major bleeding events, which have also been observed with clopidogrel consumption. However, further studies with larger sample sizes are required to attain more definitive results and precisely determine its efficacy and safety.

## Conclusion:

The most important findings of one month ticagrelor consumption in this series were dyspnea (28.8%), bleeding (2.5%), and hypersensitivity reaction (0.6%). No case of bradycardia and stent thrombosis was detected. In our study , iranian population has more susceptibility to dyspnea than PLATO result. The rate of drug discontinuation in this series of cases was 6.4 %.

## References

[1] Park J, Min J, Kang J, In Y (2015). Acute myocardial infarction due to stent thrombosis after administration of intravenous epinephrine for anaphylaxis. Chinese medical journal.

[2] Tyczyński P, Karcz MA, Kalińczuk Ł, Fronczak A, Witkowski A (2014). Early stent thrombosis Aetiology treatment and prognosis. Postępy w Kardiologii Interwencyjnej= Advances in Interventional Cardiology.

[3] Rademakers L, Dewilde W, van de Kerkhof D (2012). Early double stent thrombosis associated with clopidogrel hyporesponsivenesss. Netherlands Heart Journal.

[4] Olechowski B, Ashby A, Sambu N, Mahmoudi M, Curzen N (2016). Stent thrombosis patients with hyporesponsiveness to clopidogrel, prasugrel, and ticagrelor: a case series using short thromboelastography. Case reports in medicine.

[5] Jariwala P, Bhatia H, Kumar EAP (2017). Sub-acute stent thrombosis secondary to ticagrelor resistance—Myth or reality!!. Indian heart journal.

[6] Fan Z-G, Zhang W-L, Xu B, Ji J, Tian N-L, He S-H (2019). Comparisons between ticagrelor and clopidogrel following percutaneous coronary intervention in patients with acute coronary syndrome: a comprehensive meta-analysis. Drug design, development and therapy.

[7] Volney C, Collins A, Adams S (2019). Ticagrelor versus clopidogrel in the management of acute myocardial infarction. Journal of community hospital internal medicine perspectives.

[8] Husted S, Van Giezen J (2009). Ticagrelor: the first reversibly binding oral P2Y12 receptor antagonist. Cardiovascular therapeutics.

[9] Dimitrova G, Tulman D, Bergese S (2012). Perioperative management of antiplatelet therapy in patients with drug-eluting stents. HSR proceedings in intensive care & cardiovascular anesthesia.

[10] Akhlaghi A, Shirani S, Ziaie N, Pirhaji O, Yaran M, Shahverdi G (2011). Cytochrome P450 2C19 polymorphism in Iranian patients with coronary artery disease. ARYA atherosclerosis.

[11] Reejhsinghani R, Lotfi AS (2015). Prevention of stent thrombosis: challenges and solutions. Vascular health and risk management.

[12] Bergmeijer TO, van Oevelen M, Janssen PW, Godschalk TC, Lichtveld RA, Kelder JC (2018). Safety of Ticagrelor Compared to Clopidogrel after Prehospital Initiation of Treatment. TH Open.

[13] Goel D (2013). Ticagrelor: The first approved reversible oral antiplatelet agent. International Journal of Applied and Basic Medical Research.

[14] Wallentin L, Becker RC, Budaj A, Cannon CP, Emanuelsson H, Held C (2009). Ticagrelor versus clopidogrel in patients with acute coronary syndromes. New England Journal of Medicine.

[15] Wallentin L, James S, Storey RF, Armstrong M, Barratt BJ, Horrow J (2010). Effect of CYP2C19 and ABCB1 single nucleotide polymorphisms on outcomes of treatment with ticagrelor versus clopidogrel for acute coronary syndromes: a genetic substudy of the PLATO trial. The Lancet.

[16] Levin L-Å, Wallentin L, Bernfort L, Andersson D, Storey RF, Bergström G (2013). Health-related quality of life of ticagrelor versus clopidogrel in patients with acute coronary syndromes—results from the PLATO trial. Value in health.

[17] Kang H-J, Clare RM, Gao R, Held C, Himmelmann A, James SK (2015). Ticagrelor versus clopidogrel in Asian patients with acute coronary syndrome: a retrospective analysis from the Platelet Inhibition and Patient Outcomes (PLATO) Trial. American heart journal.

